# Covid 19 - some Lessons from Public Administrations for Humanistic Management

**DOI:** 10.1007/s41463-022-00125-5

**Published:** 2022-03-24

**Authors:** Renato Ruffini, Valerio Traquandi, Marta Ingaggiati, Giovanni Barbato

**Affiliations:** 1 Department of Law, University of Milan, Milan, Italy; 2 School of Language Mediation and Intercultural Communication, University of Milan, Milan, Italy; 3 Department of Social and Political Sciences, University of Milan, Milan, Italy

**Keywords:** Public management, Covid 19, Relational management, Crisis management, Humanistic management

## Abstract

In order to understand how the logic of public management can enrich humanistic management’s practices, the current paper will analyze the managerial practices adopted by public administrations within a situation of emergency, a condition where the specific features of the public management can emerge more clearly. Specifically, it will focus on the ways in which the municipality of Bergamo (one of the hardest-hit cities) have reacted to the Covid-19 pandemic, outlining interesting managerial practices especially from the point of view of Humanistic Management’s theory. Such interest resides also in the fact that although the Humanistic Management’s field of research has dealt with a wide range of topics (including human development, emancipation and progress), so far, however, it has not yet considered public administrations, whose role is by definition oriented towards human development through the creation of public value. The analysis of public management through the lens of Humanistic Management can be useful in various respects. Above all, the difference between public administrations and private enterprises can also lead to a very much different process of value creation, based on collaborative forms of production as well as relational and reflexive forms of management. In accordance with the Humanistic Management framework, also business organizations must generate social wellbeing. From this point of view, the lesson of public administrations can be extremely useful for business organization and management alike.

## Introduction

When the pandemic started - more than two years ago - it was undeniably difficult to find a single positive effect. The advent of COVID-19 has put a strain on both societies and people, also due to the curfew and the strict measures implemented to avoid contagion. In such an emergency, a key role was played by the public administration, for its ability to continue to provide services to citizens, to respond to increasing environmental complexity by mobilizing and coordinating also other types of stakeholders (businesses, volunteer organizations, private citizens). This role is of considerable interest because the public administration has reacted to pandemics with managerial practices that can be read through the lens of humanistic management. The ability of the public administration to adapt to a society that continues to change is undoubtedly important for both policymakers and practicing managers, but also for scholars of humanistic management.

The Humanistic Management’s field of research deals with a wide range of topics, focusing on processes of human development, emancipation and progress. In the last decade, the issues that have deserved special attention were those regarding the notion of human dignity (Bal 2017; Kostera and Pirson 2017; Pirson 2019), sustainability (Ferguson et al. 2019; Sasse-Werhahn 2019), freedom (Dierksmeier 2018; Pirson 2018), business education for a more holistic understanding of management (Amann et al. 2011; Dierksmeier 2016; Lepeley et al. 2016), social innovation (Fisk et al. 2019) and agapic love in management (Sferrazzo 2021). These works had been followed by in-depth studies on more specific topics, re-interpreted through the humanistic paradigm, which can be summarized as being orientated towards the protection of human dignity and the development of societal well-being.

So far, however, the Humanistic Management’s perspective has not considered public administrations, whose role is by definition oriented towards human development through the creation of public value. Such concept can be understood as the achievement of human progress in the public sphere through the pursuit of societal goals as defined by the democratic institutions of territorial political communities (Benington and Moore 2010; Moore 1995).

The analysis of public management through the lens of humanistic management can be useful in various aspects. In particular, the different nature of the economical processes developed by the public sector, in contrast with that of private enterprises, leads also to a very much different process of value-production, where the public administration can offer interesting insights to the private management regarding the processes of value creation, in view of the multiple goals given by the need of balancing the interests of all stakeholders. In the public sector value is generated by promoting human progress and social wellbeing, through the provision of different public services such as regulation, distribution of financial resources etc… which are both technically and socially sustainable. According to Sen’s approach on capabilities (Sen 1987, 1999), public policies and interventions should focus on what people are able to do and be, on the quality of their life, and on removing obstacles so that they are better able to live the kind of life that they have reasons to value (Robeyns 2005).

Following the same logic, value-creation can be implemented in businesses by constantly reconciling the needs for the production of wealth generated from market exchange with those of equity and social sustainability. It is indeed limiting to assess the value generated by firms by just looking at the profits because, as pointed by Knight (1923), while the output of a firm is mainly measured in terms of its price, this, however, does not completely correspond to its ethic value.

Therefore, the underlying idea of this article is that the typical form of creation of public value implemented by public administrations can help private firms to incorporate logics of humanistic management in their managerial practices, by also providing specific approaches centred on relational and reflexive management. Only by fostering and supporting relationships, dialogue and cooperation, both internally (among managers and employees) and externally (with clients, providers and the society in general) it is indeed possible to create the long-term conditions through which people can flourish.

In order to show how the logic of public management can enrich humanistic management’s practices, we will analyse the managerial practices adopted by public administrations within a situation of emergency, as such condition more clearly emphasizes the specific features of the public management. Specifically, we will investigate the approach adopted throughout the Covid-19 pandemic by the municipality of Bergamo, one of the cities most deeply affected by the first phase of the pandemic worldwide.

We hope to offer valuable contributions to business organizations and management by outlining how public administration can improve the theoretical assumptions of managerial theory by shifting to the logic of humanistic management. The shift from rational practices to relational and reflexive ones, as well as the development of collaborative networks between staff and stakeholders to answer the problems raised by pandemic, allowed the management to create value in a situation of crisis. Hence, collaborative and co-productive organisations may increase their social responsibility, and even creating public value for their community. Furthermore, we believe we can fill the gap of humanistic management literature, by applying humanistic precepts to public administration.

The rest of the paper is structured as follows: in the next section, we discuss how the concept of public value can enrich the humanistic management approach. We then proceed to illustrate the main features of the relational and reflexive management approach, while the fourth section discusses how covid-19 affected the practices of public administration. Sections 5 and 6 provide the methodology and the description of the case-study (Bergamo municipality), highlighting how the municipality reacted and faced the pandemic by fostering a relational and reflexive approach of management. The case is presented through a narrative approach, describing the three subsequent stages of the crisis. Finally, we conclude the paper outlining the lessons that public administrations can learn from the creation of public value in an emergency period.

## Humanistic management and public value

The concept of public value can be a useful tool also for private businesses, if applied as a modus operandi to its management as well as to the relationship with the external environment. The first framing of the concept can be traced back to Moore (1995), as part of the idea that value is created by the investigation of public managers (the actual explorers of value), who strive, with a pragmatic and non-ideological approach, to improve the social outcomes through the production of services. These would be: contextually and objectively useful to the beneficiaries; sustainable within the internal organization; and, lastly, legitimized by a significant consensus among the different social actors that are external to the organization. Such a strategic triangle, which is crucial for public administrations, at a closer look is revealed to be a necessary condition for also many enterprises, which must make similar evaluations for their strategic decisions, and in particular they must take into account social consent. There is, in a few words, a reference to a concept of value that shows two main features. The first one is the acknowledgement that “value” refers to a wide range of responsibilities that should be operating not only with reference to the present, but also to the future, to the future generations’ interests. From the viewpoint of private businesses, this also means that the product being sold through the market exchange must be also morally acceptable and sustainable in a long-term perspective. The second characteristic is that the concept of value is not a static one, but it takes shape through concrete activities of production of services and through the debate on their appropriateness in reference to principles of legitimacy, trust, and social justice. The creation of value is thus achieved through debate, partnerships and co-production processes that involve the different social agents. This has become particularly visible in the way in which public sector organisations strive to confront “wicked problems” such as immigration, refugees’ management, and poverty (Geuijen et al. 2017).

Similarly, businesses need clients’ approval in order to survive, and their competitive advantage can be indeed improved through the creation of strong relationships with the latter, e.g. by involving them during some of the stages of the production process, as it is underlined by the flourishing of phenomena like that of sharing economy, as well as by an increasingly common shift from consumer to prosumer and co-producer.

Therefore, the creation of public value naturally entails a strong relational ability by the public management - in other words, the ability to identify the best possible balance among all those different stakeholders’ needs that constantly emerge from cooperation and dialogue, both internally (relationship between managers and employees) and externally (relationship with its main stakeholders). The following section will thus discuss the core features of the relational and reflexive logic in public management, which will be empirically illustrated through the case study chosen.

## Relational and reflexive logic in public management

As previously mentioned, the creation of public value, that is of outputs that are not only economically relevant but also morally and socially sustainable, entails that the management of any type organisations acts in a relational and reflexive fashion. This approach should be intended as a complement to the classic managerial approach based on a rational logic (Cunliffe and Jun 2005), whose main features are the reliance on a technical-based judgment and a profit-based focus. Moreover, a rational logic necessarily implies that the environment can be fully and comprehensively understood, analysed, and anticipated in its evolution, However, a logic based on absolute rationality of decisions and the economic optimization of choices might overlook the need to balance multiple and heterogeneous needs/interests that often-characterized public interventions in fields such those of health, education and urban planning, especially during the implementation stage. By contrast, these can be better addressed by introducing a rational logic with a relational and reflexive logic as will be discussed below.

In order to fully understand the distinction between rational and relational logics, it is relevant to illustrate the distinction between calculative thinking and meditative thinking illustrated by Heidegger (1966). Calculative thinking is proper of the scientific and professional activity, and thus of a rational logic, and ‘aims at closure and categorization as a means of understanding objects and situations - a form of thinking that does not question the assumptions underlying actions’ (p. 88).

On the other hand, meditative thinking, which is at the basis of a relational and reflexive approach, does not imply an impeccable depiction of reality but is more concerned with an ongoing ‘act of questioning the basis of our thinking, surfacing the taken-for-granted rules underlying organizational decisions, and examining critically our own practices and ways of relating with others’ (p. 88). Therefore, meditative thinking is not finalized in developing quick solutions, but it remains open to the uncertainty that comes with discussion and confrontation in order to develop shared opportunities of action (Cunliffe and Jun 2005).

From a more operative viewpoint, the relational approach is based on three aspects of mutual interrelation (Gittell and Douglass, 2012): *relational leadership*, regarding the relationships between managers and employees, *relational coordination*, regarding the relationship among workers, and *relational coproduction*, regarding the connections between workers and users/clients. The approaches adopted by those who work according to reflexive and relational logics are characterized by (Cunliffe and Eriksen 2011):

- The creation of an environment where mutual knowledge and the definition of shared partnerships and visions is promoted in order to value appropriately the role and work of everyone within the organisation.
- Accepting and confronting with the internal differences by striving to balance all the interests. This is done through an ongoing dialogue in which the differences are seen as resources through which new options and opportunities can be explored.
- Creating symbolic moments that allow to work out the differences without conflicts, for example by introducing some “liminal spaces” able to foster socialization and a shared communion among workers (Van Gennep 1960; Turner 1974, 1982).
- Understanding the importance of relational integrity, and therefore acting in a reliable and responsible manner when dealing with others.
- Paying appropriate attention to the surrounding environment and to the others while avoiding self-referentiality.

Gittell (2006) has indeed claimed that relationships between shared goals, shared knowledge, and mutual respect tends to promote frequent, timely, accurate, and effective communication, further increasing participants’ attentiveness to the situation and to one another.

## The Effects of Covid-19 on practices of public administrations

In order to understand the contribution of public management to the development of humanistic management, following the logic outlined above, it can be useful to reinterpret the impact of Covid-19 on the system of social and economic relationships. As always happens, the macro and micro experiences of a tragedy provide with the opportunity for a deeper understanding and a reinterpretation of the social systems we are embedded in, as well as opening up the space to new visions of management. It is within such frame that the analysis of the public administrations that were able to confront the epidemic with an adequate efficacy can teach us about the organisational and managerial logic that allowed them to retain a sufficient level of resilience and of ability to respond to the new social and environmental complexity. These are the very situations that, to the eye of an attentive observer, can highlight the practical methods through which new forms of management, more aligned with a humanistic perspective, are applied. Such methods can easily become the rule also in situations characterized by a lesser complexity and exceptionality, as well as in a variety of contexts where more classical and rational forms of management are normally applied. In particular, there are three problematic aspects that Covid-19 has highlighted, triggering significant modifications in public management. The first one relates to the inequalities generated by managerial practices. The second and the third relate to a shift from a rational to a relational logic of management, directed both outside, to the stakeholders, and inside of the organisation, namely to the employees.

The first aspect is related to the ever-growing awareness that managerial practices and the work structures of enterprises are a source of inequality, and regarding such topic, a wide consensus has been reached about the need of protecting the subjects who are at a higher risk of marginalization. The public administrations of every country have taken responsibility for dealing with the main inequalities generated by the pandemic (Deslatte et al. 2020; O’Flynn 2020). It has been a long time since the management sciences have discovered how managerial practices can contribute to inequality, and on this topic, we can now find abundant literature, especially on issues relating to salaries and assignments. However, such analysis usually remains within the internal limits of the organization and of considerations on management techniques. In reality, the modalities in which organisational practices gets entangled with social inequalities are very far-reaching and complex, as they often operate in an indirect and hidden fashion. There is hence a growing need to understand how organizations can contribute to inequalities in beneficial or harmful ways (Bapuji et al. 2020).

Within the processes of work-design further inequalities are generated, and we can notice how the Covid-19 pandemic has surely had different implications according to the job’s characteristics, in terms of the protection of both salaries and health. In general terms, some “performing elites are able to work from home, stock up on supplies, and practice social distance” (Reeves and Rothwell 2020), others are not. Some jobs have been particularly exposed to health risks (Italy has witnessed a sharp increase of workplace deaths due to Covid-19, particularly among doctors and nurses). Some jobs have been suspended and employees made redundant, some others have not. Some workers had to bear the costs of carrying on with their work in such difficult conditions, others had not. Beyond Covid-19, such inequalities have always existed, though have often been taken for granted and considered inevitable.

Against the inequalities emerged during the pandemic, public organisations implemented three different paths to action. The first one was to avoid the issue altogether, an approached based on a radical interpretation of the principle of individualistic freedom which tends to neglect the social dimensions of human relations. This is the case of countries where, according to the liberal ideology, inequalities are basically a problem of individual responsibility where public administration’s intervention is something that should be always limited (Ren 2020).

The second option was that of supporting citizens through the direct provision of services (supply of masks, accessible health care etc…). This approach is typical of several European countries that recognize and value public intervention through a relevant welfare system following a top-down and rationalistic approach that balances costs and benefits without a real and bilateral dialogue with citizens.

Finally, the third path is represented mainly by the approach of local public administrations. The higher proximity to the emerging issues allowed them to enact pragmatic solutions, starting from the dialogue both with the internal employees and with the citizens, which has often led to the development of co-productive solutions.

Regarding the first and second option, the concept of public value is reduced to the mere assessment of economic aspects or, at most, to the relationship between the costs and benefits of public intervention. By contrast, the third option refers to a concept of public value that is more coherent with the development of human capabilities through a relational and reflexive process, and thus aligned with the logic of humanistic management. The literature has indeed highlighted how, during the pandemic, the most effective skills of the public management were indeed the relational skills, those of stakeholder engagement and storytelling, as well as the ability to develop and foster collaborative networks (Van der Wal 2020). Moreover, the pandemic has underlined the importance of interpersonal relationships, in particular those based on mutual care, confrontation and dialogue, as an instrument of de-bureaucratization of several administrative activities.

## Methodology

In order to show how relational and reflexive practices have been developed and fostered during the Covid-19 pandemic, the municipality of Bergamo has been chosen as an exemplary case-study. The city has indeed become the symbol of the global tragedy generated by the pandemic through iconic images like those of military trucks moving the deceased to other cities’ crematoriums. To give an idea of the magnitude of the tragedy, it is particularly significant to note that during the period between the 20th of February and the 31st of March, Bergamo’s death rate has witnessed an increase of 568% if compared to the average of 2015–2019. Such a case was selected because it is attuned to theoretical sampling consistent with the aim of theory-building (Eisenhardt 1989). In particular, the choice of such case provides the opportunity to show how the municipality has dealt with such a major crisis and the subsequent and delicate phase of “renaissance”, and it also allows to study the phenomenon of public value creation in connection with innovative forms of Corporate Social Responsibility for businesses.

The data has been collected through a diary method approach (Spowart and Nairn 2014), that allows participants to record their emotions and their experiences within their natural context, capturing “life as it is lived” (Bolger et al. 2003). The idea of a diary stemmed from the municipality itself, who aimed to create a collective “Crisis and Renaissance diary”, involving all the managers of the organization. The emotional intensity of the Covid-19 pandemic (both on a social and personal level), as was lived by the employees of the municipality of Bergamo, has led them to share their emotions in a collective diary of the events that have occurred since the beginning of the pandemic. The diary was uploaded on a digital platform and every manager, whenever he or she wanted, could share an idea, an emotion, a memorandum, or reporting a meaningful newspaper article. 24 managers participated in the project, which refers to the period spanning from February to September 2020.

The diary was subsequently sent to one of the authors, directly involved in the Municipality of Bergamo for many years, who reorganized the flow of thoughts. Such diary method has allowed to record the emotions of the participants and their variations over time (Beal and Weiss 2003), details which they probably might be less inclined to reveal through direct interviews with a researcher, considering how sensitive the moment was. In addition to the collective diary, the material used for this study includes the findings of four years of direct observations within the municipality, carried out by one of the authors as part of a larger research on processes of de-bureaucratization (still in progress). Direct observation has been particularly useful in order to identify the specific managerial practices, patterns of behavior and the relative influencing factors that refer to the rational, relational and reflexive logic as described above. The case has been analyzed through a narrative approach, which maintains that people make sense of their own experiences through the narration of the same (Bruner 1991; Czarniawska 2004; Rhodes and Brown 2005; Sims 2003), or in other words, that it is through the narration of their own experiences that individuals express the emotions they have lived through, while the recollection of the story identifies a narrative strategy which makes sense of and rationalizes their lived experiences (Ellis 1991; Fineman 2004). Qualitative content analysis (Miles and Huberman 1994) has also been used to identify words and sentences that refer to elements, practices and behaviors proper of the relational and reflexive logic, as those illustrated in Sect. 2. While one author was the main author responsible for this process, the results have been cross-checked by the other two authors in order to reach an agreement on the coding output and increase its reliability. This approach allowed to highlight the different stages through which the relational logic has emerged, and as a result, the case-study is arranged into three different temporal phases. The beginning phase coincides with the emerging of the crisis, along with the difficulty in grasping its full dimension, followed by a subsequent realization and a shift to a more conscious response. Describing this phase, we provide a framework of the pressing issues, in the first response to the emergency. Lastly, a brief description of what the Second Phase of the pandemic is presented, known as the phase of the “Renaissance”. The diary alternates the recollection of the events with reflections on their deeper implications. Some quotes from the diary are also presented in order to give some sense of the original nature of the manuscript.

## How the Municipality of Bergamo has confronted with Covid-19

### The beginning

The city of Bergamo has witnessed an unprecedented period of development in the past 6 years: tourism grew by 60%, cultural initiatives have multiplied and the municipality has quickly climbed the rankings for quality of life and innovation. The public works have encompassed all districts, and some of those parts of the city that have been standing still for decades have been finally “rehabilitated”. Bergamo was experiencing a magical moment but, suddenly, COVID arrived, and shortly thereafter, it would have become one of the areas with the largest number of Covid-19 infections globally.

On Friday 21st February 2020, a few days before the proclamation of lockdown, the managers of the municipality were experiencing a relational moment, an example of what we called *relational leadership*. The *officina*^1^, a collective training activity involving all the managers and the administrators of the municipality, has with the time had turned into a sort of ritual managed in a liminal fashion, as it takes place at different times and places from regular work (despite being a moment of work), a moment when people meet each other as people rather than colleagues.

Traditionally, in those moments, *“a group of managers will cook for everyone, led by a starred chef who has been supporting these moments of sharing for years, as in the previous officina”*. The same was happening on the 21st February, when suddenly, during the group activities, the information about the cases of Coronavirus in the Codogno area (in the province of Lodi) began to circulate. The terrible disease that in the previous weeks it had seen spreading in China was now arriving in Italy. At that moment, also a hint of fear began to peek out. From Saturday, everyone at home followed the information that gradually appeared on social media and online newspapers.

*The picture became more and more serious, and so the news. The feeling that it was not such an easy problem to deal with was reaching to us. It was the first weekend when we began to send each other many messages outside the “canonical” working hours. A habit that became commonplace in the following weeks. The “Moloch” of working hours was beginning to waver.*

In the following days, a shifting climate began to establish itself, made of a spasmodic wait for data, figures, and information. A search for certainties in a sea of uncertainties that were gradually mounting. In the early days, the hope was that it represented a manageable phenomenon, and the slogan was “the city does not stop”: this was a consequence of the refusal to interrupt the daily routine because of the fear of dying or getting sick. Just a few days were enough though to realize that the municipality, its employees and the citizens were facing a much more serious, more widespread and pervasive phenomenon than the infections of previous years, and that, in reality, little or nothing was known on how it would have evolved. In a few days there would be a lockdown, but the municipality would still have to be able to provide with its services.

### The first phase. Nothing is a matter of chance: relations as a way to handle the virus

The ways in which the municipality managed to organize itself effectively in such extreme conditions did not happen by chance, but because there were preconditions and habits already in place for several years: *“Many of the things we did and the results we achieved would not have been possible without a network of positive relationships, built and nurtured over time when we were NOT in an emergency situation”.*

From the diary analysed, we found some concrete examples of relational co-production practices, especially in regard to the mobilization of volunteers and the management of the first emergencies.

#### The case of volunteering

Regarding the case of volunteering, in the municipality *“the volunteers’ service network was immediately set in motion: various associations decided to collaborate, and the Alpine troops were also mobilized to build a field hospital with intensive care in just 8 days. Our municipality looked for 500 volunteers available in a short time, but 1000 volunteers in the following 2 weeks responded”*.

The enthusiasm in the response received was founded on some crucial preconditions. Beyond the civic sense of the citizens of Bergamo, something had long been sown. For some time, the municipality had initiated practices of co-planning and co-production of services, where the rules are malleable and constantly negotiated. In practice, the municipality had very flexible arrangements with local associations, and a strong presence of social networks and neighbourhood operators. The latter have, in the past 5 years, completely revolutionized the organisation of districts and neighbourhoods, shifting from a logic of political representation to a logic of social participation. Because of this, as soon as the hardest phase of the pandemic began, the volunteers’ network immediately set itself in motion from every corner of the city. This time, even more than before, there was also the help of many single individuals, not related to any formal associations or structured networks.

*While we tried to understand what were the tasks that volunteers could help with, we also understood the ability to do something for others could as well work as a form of healing for the volunteers themselves. Many times in recent months we had theorized the idea that the distinction between those who “heal” and those who are “healed”, between those who provide a service and those who receive it, was now showing its limitations.*

#### The response to the emergency

Co-production practices appeared not only among citizens, but also within the institutions. The preconditions that were established in the recent years had facilitated the creation of strong bonds between people in the organization as well as between institutions. These kinds of relationships are claimed to prevent public institutions from the risk of becoming a bureaucratic iron cage (Gittell & Douglass, 2012).

The reference to the “response strategy” leads to observations that are far from being purely axiomatic. The same need can lead to different response strategies. One path is to promote a wide synergy among different actors, professionals as well as volunteers, in order to define a complex and integrated system of actions, while allocating the resources found both internally and externally on the basis of a shared project. In this case it is necessary to resort to tools and procedures different from those that govern the purchase of services. One example is co-planning, which, despite having until recently been seen with a certain suspicion by the administrative apparatus, it is now fully legitimized by the Constitutional Court and subsequently by the parliament. Hence, now that the doubts that relegated the collaborative practices to a residual position (when compared with the competitive ones) have been progressively dissolved on the normative front, the game becomes effectively a political one, and it will be easier to convince the town clerks about the feasibility of co-planning. At the same time, a new perspective has emerged, stemming from within the Third sector as well as from the dialogue with the political representatives of public administrations: a rationale that does not start from the preference for a specific means, but from the framing of the responses to the needs through subsidiary and collaborative strategies. From such a change a cultural and organisational challenge emerges, which is unprecedented for those social enterprises to whom, for the past 25 years, the market has asked to adapt in order to respond to a purely competitive context. They are now called to reinvigorate an original vocation, never eliminated but in some cases necessarily relegated to the background. Today is the very Procurement Code that testifies how a certain vision has been overcome. The choice between tools based on competition and those based on collaboration is hence a consequence of the nature of the need that the administration identifies: to stock on services at the best conditions of quality/price, it will make use of competitive tools; to promote widen synergies and integration among various actors towards a shared goal, it will opt for collaborative tools. There is no ex-ante planned strategy, but only the will of the administration to identify the strategies more suitable to respond to the emerging needs.

The emphasis on collaborative tools has been the outcome of a consolidated tendency in the strategies of social intervention which aims at building *“integrated systems of interventions and services”*, and hence at creating synergies and collaborations, rather than promoting the mechanisms typical of market competition (without however denying how, in many situations, the latter can be proved to be functional to the realization of appropriate services for the citizens) (Adler 2001). An example of such process were the local shops, prompted to provide home delivery services. And as soon as the municipality got few facemasks, it used them in a targeted distribution in favour of the higher-risk categories. Also in this case, to have previously managed a call for bid to obtain the shops’ availability to provide “proximity services” in the neighbourhoods, and to have nurtured positive relationships with the merchant associations and the neighbourhood network operators, have all been fundamental preconditions.

The management of fatalities is another example. This is a world apart, normally left to its own routines. But in this situation, it became a crucial and very delicate aspect to manage. The total deaths for the entire month of March were about 8 to 10 times higher than the average. Consequently, the rate of cremations had become unsustainable.

*We had to act quickly, and we immediately made two decisions: to close the cemetery to the public and to prepare the church to house the coffins awaiting cremation. This has been a difficult choice that profoundly limited the lives of some elderly people who have a visit to the cemetery as their daily and central activity*

The graveyard and the “All Saints” Church are owned by the municipality. Yet, this is still a church because is managed by capuchin friars: managers tried to take advantage of the excellent relationship that the municipality retains with the local bishopric, which has for many years been actively engaged on a variety of social projects. Thanks to such relationship, the church was made available spontaneously without having to recur to administrative decrees. Such an approach has been then also applied by the other neighbouring municipalities which, as the days went by, have found themselves dealing with the same problem (also because, sadly, the cemetery’s church filled up in an alarmingly short time, with more than 100 bodies awaiting). The problem then became that of increasing the potential of the crematorium. On this issue, the fact of having reviewed and modified the concession in recent years in a way that enabled more flexibility, proved itself to be the most useful. Through such adjustments, the possibility of doubling the production line was introduced, along with the facilitation of 24/7 operations and a flexible financial system able to adapt to the variations in demand.

*The introduction of these modifications had to overcome some resistances, of the kind that often follows innovations, but with patience and determination we reached our goal*

However, when the number of the deceased grew further, even such a move proved to be insufficient. Managers then asked themselves whether to implement a decree that would force the burial even on those who had requested cremation. Formally it was a viable path, but they chose to avoid it in order not to hurt the sensitivity of the relatives of the victims. The only other way left was to find other cremation facilities. Here the collaboration with the police force started, who acted immediately without even wondering what role they were acting in, if on behalf of public order (which has to deal with gathering and protests of the relatives of the deceased, prevented from participating to the funerals) or as a response to a sanitary emergency (the permanence beyond 10 days of corpses in the funeral parlour can be dangerous), or to a wider emergency for the community. Managers immediately started to work side by side in order to find available cremation facilities. The numerous positive relations established with many other medium-sized municipalities also proved to be effective. In view of an unsustainable workload for the workers of the funeral parlours, and thanks to the relationship with the municipality of Trento, an agreement was reached with the clerks of the city’s parlours who would be hosted in hotels and would support the local facilities. Such relationships between municipalities allowed to activate practices that do not necessarily pertain to their formal responsibilities, but were nonetheless made possible thanks to the general role that the municipality plays on the territory. Thanks to such synergy, the structures were easily found, and within 24 h the logistic support of the army for the transfer of the remains was obtained. To such aim, a procedural stretching was implemented, that is when standard and normal procedures are temporary put aside in favour of temporary and ad-hoc solutions. Within 48 h the municipality also managed to create a public fund that enabled the funeral directors to advance the payment of cremations outside Bergamo, in order not to burden the victims’ relatives further.

*We, as managers, stopped “marking our territory”: everyone has rowed in the same direction and has welcomed the directives, even when not completely convinced by them, as we understood that in such moments if we do not march together, we will not arrive anywhere. We forgot about the concept of “ownership” of the specific resources assigned to each sector (being them people, expenditure items, skills etc…). We went from “mine” and “yours” to “ours”! We realized that we belong to a dendritic organization defined by mutual connections*

The relational routines enacted, along with the respective operational practices and affective relationships, made it possible to respond to the emergency and enable a rebirth: hence, looking at Bergamo’s reality, *“everyone could appreciate how the alpine units have managed to realize an emergency hospital with an intensive therapy unit in record time, how the medical staff have made miracles to respond to the emergency in hospitals and on the territory, how doctors and nurses have come from all over the country to offer their services, and how thousands of civil volunteers have engaged in the realization of a municipal solidarity network, (and much more…). And also for a lot of us from within the administration it came naturally (as if it was taken for granted) to act in support of the struggling cemeterial services, to implement the coordination of civic volunteers aimed at facilitating the lockdown, to organize the smart-working networks and hence being able to move easily and quickly into a flexible working mode”*.

Confronted with the question of why this kind of behaviour has been naturally and voluntarily adopted, the recurring answer is of a disarming simplicity: *“because it was necessary, it was the right thing to do!”*. From such an answer, it becomes evident that the need to act had prevailed on the issues on *how* to implement such actions. This is not what happens in ordinary circumstances.

#### Care in connecting: distant but close

The need to guarantee social distancing meant that remote-working became the ordinary way of working. Bergamo activated the remote-working since the very begging of the emergency, also because the municipality was one of the local administrations that had already developed this approach. However, suddenly, the number of employees working in such a mode jumped from 30 to 440.

*“In our normal idea of life, such practice occupied the time of everyday life in balance with a free time rich in social relations and commitments, and, especially, open to the outside world. Within an emergency, it is very difficult to find the balance highlighted above, and indeed we realized that the habit of entrusting somebody’s attention to their own work becomes pervasive, almost as if it automatically fills in the spaces left empty by the increased lack of sociability”.*

The paradoxical aspect of distance the emerged is the need to see each other that comes with it. When employees could see each other more easily in person, they mainly used e-mails, or at most phone calls. The e-mail, and sometimes even the telephone call, are limited communication methods and lend themselves to distortions. The e-mail does not allow you to hear the voice that makes you understand the real intention of the text, nor the face that further characterizes it. The excessive use of e-mails has many times created ambiguity (how many e-mails misinterpreted or sent “at the attention” of an unnecessarily large audience that only obtained the effect of fomenting the “hooliganism” in support of one position or another…).

*With the COVID emergency we discovered video calls. The absence of the face, previously so obvious, prompted us to use a method of communication more effectively than before, when we could actually see each other.*

In this way, remote-working has become a perfect example of what we mean by “relational coordination”. To emphasize this is worth noting that such need to see each other, to be together, and to support each other led almost spontaneously to the organization of a remote version of the officina workshop. Initially, it seemed impossible to achieve, because one of the added values of the workshops was in fact that of being physically close, of having dinner together: in rituals, the physical proximity of the bodies is always an essential element. However, on the 8th of April, an online version of the officina took place: managers and administrators were connected online, a psychologist was present to help understand and confront with the pain, and not to go through it alone.

*We told each other what we were seeing and experiencing, and then we organized group activities, obviously online. We knew the method, we had been practicing it for 6 years, so we distributed the tasks among the different groups, namely: to identify the significant individual and collective episodes, to find which organizational mechanisms were enacted, to analyse the formal and regulatory changes needed, to identify the assumptions that allowed these changes to take place in such a short time, and to reflect on the permanent effects on our way of working that we would like to see being enabled… Finally, we came back, all together again, in the plenary room, to share what happened in the groups. In an ordinary ‘officina’ we would always end up sharing dinner all together and, sometimes, with a playful or musical moment (this is also important). At first, we were not sure how to do it… but then we had an idea: let’s play an online team game that makes us smile, let’s look for a pastry shop willing to deliver a cake to the winners’ home. It was a truly exciting day.*

During the Officina, managers understood that co-presence can also be virtual, if the faces and feelings of others are identified. As underlined by recent studies on pandemic and the literature of emotional contagion, vitality can be claimed to be contagious and to spark imitation (Gibson 2020; Barsade 2002). This contagion goes beyond the screen if the relationships, as mentioned before, are inclusive and direct. That meeting, despite the situation, and in full respect and continuous remembrance for the dead, was incredibly vital and energetic.

*“We have seen again the faces that we missed, but this was effective also because we already had a certain habit of being together physically, in a way that was respectful and inclusive, not just formal and hypocritical. This allowed us to give continuity to the thin thread that binds the real things and the bits. Finally, we have been lively. We not only worked, but we also played and made jokes, creating a sense of psychological and physical energy that allowed us to face the conditions of loneliness and hardship produced by social distance, especially considering the grief that almost everyone was experiencing”.*

What has happened? Reflecting and rereading, the most convincing answer is that they have moved from “social distancing” to “care in connecting” (Gibson 2020). The care and attention involved in the (remote) connection in this critical phase were essentially based on an idea of compassion.

*“The first question at the beginning of each phone call was” How are you?”: It was no longer a simple rhetorical question, an empty formalism. Now it has become a real question, in which I awaited a hopeful answer from a person who was close to me”*.

This new modality of work has had a significant effect on the “rebalancing” of interpersonal relationships. The lockdown, on the one hand, and the use of the web as the only available vehicle to nurture remote relationships, on the other, has in fact “levelled” people’s distance from each other, both for those who previously had privileged relationships due to their physical proximity (their “circle”) and the intensity of their relationships, as well as for those that were more distant or more difficult to reach. Thus, in concrete terms, remote working has created a situation effectively facilitating the inclusion of people, by recognizing the importance and uniqueness of the individuals while cultivating the sense of belonging to a community.

*We all declare and claim our specificity, but at the same time we want to belong to something bigger*

### The second phase: the spread of relations in the “Renaissance”. New necessities for the city’s social structures

During the emergency, and in the period immediately following the restart, the municipality has launched many initiatives which would have been previously unthinkable, both in terms of contents and delivery methods. In a few months, many things have been accomplished, things that perhaps would not have been achieved or would have taken years to. The “Renaissance” of the municipality has been made possible by numerous relationships and co-production practices established with other institutions and actors, as banks, municipalities or foundations. These relationships impact several domains, such as those concerning economic, social, and sustainability issues.

A fundamental challenge concerns the microeconomic fabric of the city, because many realities (as small commercial and professional business) face great financial challenges. In order to support them, the municipality has decided to exempt them from paying certain fees and allowed cafes and restaurant to expand their activities using public spaces.

*“We are planning a mentoring and advice service to support the businesses in the reopening. We are trying to obtain fundings to give them non-reimbursable to support the expenses necessary to reopen: from the purchase of protective devices, to the purchase of equipment and services to prevent gatherings and guarantee distancing, for the rental of the premises, for the energy bills in the fixed part etc.”*

In addition to the assistance to economic operators, in June the municipality activated the *Renaissance Fund* – a mutual-aid fund for the small businesses affected by the virus, financed by the municipality along with a bank and various other foundations.

In terms of the evolution of the welfare, for example, the project WILL (Welfare Innovation Local Lab) was launched, namely a path designed to experiment, compare and systematize practices capable of deeply innovating the Italian local welfare; a project made sustainable and suitable to the real needs of local communities. It represented a collaboration between 10 Italian municipalities that were willing to enact a bottom-up reform of the municipalities’ welfare system. The Municipalities involved have decided to cooperate in order to individuate innovative answers to structural problems that made local welfare no longer sustainable, seeking paths of interventions that respond to the needs of citizens and that are self-sustainable.

Moreover, in order to help people with disabilities and their families during the reopening phase, an integrated support plan was activated. At the same time, the municipality of Bergamo and some support groups have proposed a series of socio-educational activities, home assistance and psychological support.

In terms of urban planning, the municipality reflected along with scholars and research groups on the impact of the coronavirus on sustainable development, on the practical implications of urban planning parameters, as well as on the planning of services and transportation. Some first implementations were developed in terms of incentives for sustainable practices and behaviours.

*We concluded that we needed a (re)organization of the city's pace*: it was necessary to redefine the use of roads and public spaces, increase non-polluting forms of commuting (walking, bicycles, light mobility) and support areas that will allow for commercial, recreational, cultural and sporting developments, while respecting the physical (but not social!) distances which have been implemented.

*Above all, through our experience we realized that it is important to rediscover the dimension of the neighbourhood.*

## Discussion: Value Creation, Relational Model and Humanistic Management

The Covid-19 pandemic forced administrations to face sudden changes in their internal organization, and has highlighted important social issues, from sustainability to discrimination and much more. Both the development of organisational and managerial models, as well as the need to rethink and reinforce CSR logic, had been present for a long time already. However, the Covid-19 pandemic has accelerated the process and made evident the need to provide quick answers to the issues that emerged. The case discussed has highlighted some of the courses of action taken by a public administration that can help to define and improve the theoretical precepts of managerial theory according to a logic of humanistic management, while helping to align it to the public interest and create a better connection with social welfare creation through public value (Pirson 2017).

Following what emerges from the case of the municipality of Bergamo, we can notice how the (pre-existing) managerial approaches implemented in dealing with the crisis were of a relational and reflexive nature. Facing complexity, as well as new and unforeseeable situations, a rational approach to action is completely insufficient, and it has to be complemented with a relational activity that involves all subjects, both internal and external to the organization, aimed at finding a solution to the problems. The strongly rational approach might be also entirely insufficient in situations of extreme complexity and environmental dynamism, as it is often proven to be intrinsically ideological, and it might probably fail. Those public administrations that, throughout the pandemic, decided to act autonomously, for example by providing services and subsidies directly and exclusively through their own administrative structure, have in fact created many problems. The complexity of communication, the vast amount of the cases to be reported, the lack of scientific understanding about the pandemic, all have but generated mistakes in decision-making, which were managerial mistakes rather than political ones.

A relational approach (Cunliffe 2014) to management understands that both ourselves and the organization we work in do not live in an isolated way, but in relation to others, and that we constantly try to adapt to each other through dialogical relationships. The relational logic is related to reflexivity, in turn based on the interconnection between dialogue and action. Hence, reflexive managers should be able to critically assess how they relate to others and should be open to different visions of the world and to dialogue. In summary, according to Cunliffe (2014), relational and reflexive managers should work towards new forms of collaborative and inclusive systems. This vision is completely consistent with the humanistic management perspective, as it can facilitate the path to achieve higher human virtues among people and more effective organization (Melè 2003).

A relational and reflexive managerial model cannot be created overnight. The case above has in fact highlighted how the municipality already possessed one, and it was this very feature that allowed to find the solutions to the main issues faced throughout the pandemic, as well as to mobilize a considerable number of energies. Such a model is representative of a managerial attitude that needs to become part of the organizational culture, but very often obtains little attention as it is time-consuming and in contrast with the classic managerial logic.

Such a relational approach is developed on two fronts, the internal and the external one. From a point of view internal to the environment of the administration, the first need that has been noticed was that of caring about each other, and not only “being connected”. Such a dimension is viable even when the connection is implemented through a computer rather than physical presence. A clear case of such possibility was the experience of the online “*officina*”. In this regard, ritual collective moments can be extremely useful (Sferrazzo and Ruffini 2021).

A second dimension of the internal relationships relates to the possibility of implementing processes of de-bureaucratization, in order to counteract that process of administrative formalization that generates phenomena of organisational stupidity rather than of fairness and efficiency. Many of the simplifications adopted throughout the pandemic were surely result of the critical conditions faced, however they were identified and resolved also thanks to the relational processes that pre-existed among the different subjects (like the immediate activation of volunteers in the first phases of the crisis). Also in this case, the crisis has made the answer to a gradually emerging issue much more urgent - namely how to reconcile interpersonal relationships, crucial elements for effective administrative action, and the de-bureaucratization of systems, with the (now inevitable) mediation of the “information technology” (Gibson 2020). In practical terms, the current and future leaders will be asked to confront with the problem of exercising their leadership remotely (Antonacopoulou and Georgiadou 2021), this being but just one of the many challenges faced.

A second aspect of the relational and reflexive managerial approach concerns the relationship with external subjects. If the crisis has emphasized the need to grasp properly the relational dimension, this especially applies to public managers, whose abilities in stakeholder engagement, storytelling, handling of politics and creation and development of cooperation networks (Van der Wal 2020) are here clearly put to the test. In the case of Bergamo, forms of stakeholder engagement and co-production have been initially implemented in order to deal with the first phases of the emergency, as well as in the subsequent phase of rebirth, in order to redefine various services - like those aiming at funding small businesses, as well as the ones aimed at redesigning the welfare system that the municipality is able to offer, as well as the practices of urban planning. What we believe is worth noting is that thanks to such forms of collaboration and co-production the organizations can meet criteria of social responsibility, specifically with the creation of public value for the community through the services they offer.

## Conclusions: CSR and Public Value. What We Can Learn from Public Administration

As public administrations develop production processes that are complementary to those of private enterprises, their approach probably represents a field of study that is useful also for the development of the theory and practices of Humanistic Management (Mèlè 2003). If anything, this is due to the fact that both public and private institutions can contribute to the same goal of respecting human rights and dignity, and in general of human emancipation and progress (Pirson 2017). Specifically, the understanding of how “public value” is generated (Moore 1995, 2019) can be of high interest also for businesses. In particular, through the understanding of the mechanisms through which the public administrations operate, businesses can identify the characteristics of that kind of stakeholder management that can be useful to be socially responsible through their normal activity, namely without struggling to formalize ad-hoc CSR practices, but simply by operating within a logic of a community-shared benefit. To this end, the analysis of the ways in which public administrations (and in particular of an institution like the municipality of Bergamo) have confronted with the Covid-19 crisis can offer some potentially interesting insights. The first general feature is found in the fact that the managerial models adopted are strongly relational and reflexive (Cunliffe 2014), based on a deep engagement of the staff and of stakeholders, and using a logic of participation and co-production of services. Such a managerial approach, in itself valid also for businesses, is not however easy to be adopted by the same. In particular, the relational logic is considerably costly, and it is not certain that for the private organizations would be viable to generate public value without hindering their business model, as it is not given that they would have the ability or the interest to interact significantly with public and private subjects, namely with the community, without producing a specific value for the business. In this sense, we would probably need to investigate further the logics of production of public value that can be generated by businesses, possibly looking into the value generated by selfless actions and by agapic orientations (Sferrazzo, 2021). In any case, we believe the study of the logic of public management and of public administrations can be useful for the development of Humanistic Management.

## Footnotes

1 Since many years the municipality organizes these meetings/workshops, on a completely voluntary basis, outside formal working spaces and working hours, and involving employees with a higher degree of management responsibility. Such moments are functional not only to create occasions for the discussion of technical issues, but also to establish and consolidate deeper social bonds between the participants.
